# Hepatitis-C during Pregnancy: Antenatal period challenges, management and the way forward

**DOI:** 10.12669/pjms.40.1.7578

**Published:** 2024

**Authors:** Lubna Kamani, Asma Abdul Razzak

**Affiliations:** 1 Lubna Kamani, FCPS, MRCP, FRCP, SCE (Gastro) Professor & Director GI Residency Program Liaquat National Hospital Karachi, Pakistan; 2 Asma Abdul Razzak, FCPS (Gastro), MACG, Assistant Professor (JMDC), Medicare Cardiac and General Hospital. Karachi - Pakistan

**Keywords:** HCV, Pregnancy, Viral hepatitis, Mother to child transmission, Hepatitis-C in pregnancy

## Abstract

Pregnant women and the general public are both directly impacted by cirrhosis which is a chronic liver disease. It is also widely known that women who have a history of injectable drug use and cirrhosis are more prone to experience unfavorable consequences that have a negative impact on the health of both the mother and the unborn child. Higher maternal Hepatitis-C Virus (HCV) in pregnancy viral load, length of labor, use of amniocentesis or fetal scalp monitoring and protracted membrane rupture are all the risk of perinatal transmission of HCV in newborns. Globally, a large number of childbearing-age women become affected by HCV every year and vertical transmission of HCV is still a serious public health concern. Pregnancy-related immune alterations have a significant impact on the course of HCV infection throughout the third trimester and provide favorable circumstances for the spread of the virus. The exacerbation of hepatic damage during pregnancy and the postpartum period is mostly responsible for HCV-specific cell-mediated immune responses. An extensive literature search done via electronic search engine including Cochrane library databases, PubMed, Google Scholar, Science Direct and HCV in pregnancy articles were included.

## BACKGROUND

Hepatitis-C infection is an emerging global health challenge manifesting itself as an acute illness of a few weeks to long-lasting chronic conditions like cirrhosis, hepatocellular carcinoma, and mortality resulting from the complications of hepatic inflammation. With an 8% global prevalence, 70 million people worldwide who have the Hepatitis-C virus (HCV) experience chronic illnesses that call for transplantation to improve their quality of life.^1^,^2^ Albeit low, vertical transmission of the HCV from the mother to the child transmission (MTCT) is a significant challenge faced by antenatal and postnatal clinics globally. Major risk factors are history of multiple injections, blood transfusion and surgery.^3^ With a less than 10 percent rate of transmission, HCV during the antenatal period predisposes women to the risk of complications attributing to maternal mortality.^4^,^5^

Additionally, there is no prior information on the possibility of HCV having a negative impact on mother and child health or on the effectiveness of therapy at this crucial time in Pakistan. Much of the empirical evidence underlying HCV infections during pregnancy and its complications emerge from high-income countries where the socio-economic conditions and health systems are substantially different from that of a low-and-middle-income country (LMIC) such as Pakistan. Considering HCV being endemic in Pakistan with a high prevalence among pregnant women, this review situates Hepatitis-C infection during the antenatal period or pregnancy in the context of Pakistan to further our understanding of this infection and its management during the pregnancy period.

### Pathogenesis And Prevalence Of Hepatitis-C Virus Infection

Due to several socio-economic reasons, re-use of syringes, lack of hygiene facilities, low health literacy, and weak disease surveillance, HCV infections are highly prevalent in Pakistan with an estimated prevalence ranging from 5 to 25 percent of the population suffering from chronic complications^6^,^7^ while the estimated prevalence of the HCV infection during the antenatal period in Pakistan has been reported to be 3.27-8.9 percent.8,9 Owing to the dearth of resources, community awareness, and disease surveillance, HCV infection is endemic in Pakistan.^10^ The risk of complications and MTCT of HCV during pregnancy increases many folds in countries like Pakistan where many women only discover their infectivity status during the routine antenatal screening and medical examination. Since there is a lack of data and certain studies with extremely limited generalizability reported different prevalence of HCV infection during pregnancy ranging from less than one to 20 percent, the burden of HCV infection during pregnancy in Pakistan is not well understood.^11^

However, in recent modeling research conducted by Dugan et al., it was highlighted that approximately 21 percent of the total 70 million people suffering from HCV infection are women of reproductive age globally, of those, women from four countries in the South and East Asian region including Pakistan, India, China, and Russia.^2^ Dugan’s study findings were consistent with previous data suggesting Pakistan among one of the top four contributors to HCV viremia globally.^12^,^13^ In a local study among primigravida with jaundice, it was found that 7.5% had HCV antibodies.^14^ While parenteral routes are frequently used for HCV transmission in adults, vertical routes are mostly used for viral infection in newborns. Numerous factors are influencing this vertical transmission in the children born to mothers with HCV infection, the most significant of those are seroprevalence of HCV-RNA, human immunodeficiency virus (HIV) co-infection, and peripheral blood mononuclear cell infection.^15^

### Indications for Monitoring of Maternal HCV during the Antenatal Period

Pregnancy requires critical healthcare adherence and it is the time of great significance to screen HCV for possible earliest treatment as well. HCV screening during the antenatal period is recommended universally, specifically in pregnant women with risk factors.^16^ These risk factors, as listed in the recommendations, include a history of using illicit injectable drugs, percutaneous or parenteral exposures in uncontrolled settings, women receiving long-term hemodialysis or blood from donors with known HCV infections, women with a history of incarceration, people receiving treatment for sexually transmitted diseases, people with any type of chronic liver disease and women who have received blood from donors with known HCV infections.

However, there is a limitation to this approach for not considering the carriers without risk factors as some studies proved the presence of the virus in women without any risk factors.^17^ Out of more than 57,000 women studied in a retrospective cohort research carried out in Brisbane between 2007 and 2013, 2.5% of HCV-positive women reported with no clear risk factors.^18^ In addition, estimating the true prevalence of HCV in the pregnant population is not routine due to the lack of large-scale screening facilities as well as resources especially in developing countries. Moreover, compared to other susceptible populations, pregnant women have a far lower incidence of acute HCV, and fulminant Hepatitis-C is quite uncommon.^19^ While the rate of complications in this population group is low, there are significant variations in the prevalence of maternal HCV infections in some high-risk settings, where the screening questionnaires even failed to identify the true prevalence of HCV.^20^ In another geographic setting, the prevalence of HCV during pregnancy was estimated around (0.1-3.6)%21, whereas in the United States, a research study recorded significant escalation of HCV infection amongst mothers from 1.8 to 3.4 per 1000 live births in the period from 2009 to 2014.^22^

Considering these changing trends in the disease epidemiology, universal screening of HCV in the pregnant population is stressed upon by major health regulatory bodies across the globe as it is a feasible yet cost-effective public health intervention, as opposed to disease management and the indirect cost of care for HCV infected women and children born to these mothers.^23^ Despite these universal recommendations for HCV screening in routine antenatal clinics as part of antenatal checkups, the uptake of these recommendations in clinical practice is impaired, particularly in developing countries where health systems are weak and shortages of healthcare facilities as well as the health workforce are common.^24^ This shows that in these nations, immediate effort is required to raise education of Hepatitis-C and its detection and intervention treatment.

Reaching women who could have HCV through prenatal care as well as other women’s wellness appointments is crucial, especially for women who inject drugs or who might not otherwise be involved in the medical system.^25^,^26^ However, research on the incidence of infections in pre partum patients does not detect infections in pregnant women who do not get medical treatment.26 Moreover, preventive monitoring and therapy are crucial because prenatal HCV treatment is not clinically authorized. Due to vertical transmission, the use of injection drugs has grown among young women, which has elevated levels of HCV in both this population and in children.^20^,^27^

### Physiological Changes Attributing To Maternal and Neonatal Complications

Pregnancy is marked by a plethora of physiological changes that assist the fetus’s growth and development, also evident from differences in laboratory findings of glucose level and other biochemical markers inclusive of hepatic enzymes and hormonal profiles.^27^ Although minimal (3 to 5 percent incidence), reduction of hepatic transaminases, albumin, bilirubin, and gamma-glutamyl transferase (GGT), and elevation in alkaline phosphatase levels during pregnancy is not unusual^28^, special consideration needs to be accounted for women who have existing HCV infection has a higher risk of consequences. The alterations in liver enzymes are caused by the production of endogenous interferon from the placenta during pregnancy, although their involvement in antiviral defense during pregnancy is unknown.^29^

Other modifications such as hem-dilution or immunological tolerance were seen during an HCV-infected pregnancy, which may explain for the reduction in serum transaminases throughout pregnancy. Sex hormones and immunosuppressive cytokines are produced, which may result in a modification of the immune response to HCV in pregnant women.^21^ Evidence from clinical practice also indicates a high rate of incidence of gestational diabetes and hypertension in HCV-infected women. Reddick et al., highlighted that pregnant women with HCV infection had a higher rate of gestational diabetes.^30^ In addition, there is preterm delivery, low birth weight, and being short for gestational age, and congenital abnormalities among HCV positive mothers have more children than HCV negative mothers, which were statistically significant at <0.01 level of significance in a study conducted in the United States.27 Moreover, HCV RNA-positive mothers show a higher chance of vertical transfer of the infection to the new born. These women are also more likely to present with HIV co-infection alongside respiratory anomalies including asthma, obstructive sleep apnea, as well as gastrointestinal complications.^21^ Of all gastrointestinal complications, pregnancy cholestasis is more common in women with HCV infections, usually more pronounced during the first trimester of the pregnancy.^21^

### Pregnancy Consequences In Women With HCV -Related Accelerated Liver Cirrhosis

Chronic liver disease, which progresses to complex cirrhosis, is known to affect both the general population and pregnant women equally. It is also widely known that women with a history of injectable drug use and cirrhosis are more prone to experience unfavorable consequences that result in poor health outcomes for both the mother and the child.^2^,^3^ Though there is a paucity of data on the pregnancy outcomes of women with cirrhosis in underdeveloped countries such as Pakistan, a descriptive-exploratory study from the United States of America identified chronic liver disease as one of the six top causes of mortality among women of child-bearing age.^23^ While the impairment of hormone levels and exacerbated metabolic changes due to liver disease are attributable to reproductive health issues, it can be postulated that these hormonal impairments and metabolic changes could possibly cause adverse effects on the growing fetus responsible for fetal anomalies discussed elsewhere in this review.

Another study from the United States found that women with chronic Hepatitis-C had a greater risk of intrauterine membrane rupture.^16^ Furthermore, an increase in blood flow and intrahepatic resistance are two recognized outcomes that cause raised pressure in the portal veins, resulting in portal hypertension and the production of esophageal varices. This extremely high intra-abdominal pressure has been identified as a severe risk factor for esophageal varices bleeding, particularly as a result of repeated Valsalva maneuvers used to manage increased heart rates during delivery. Evidence suggests that the likelihood of maternal mortality due to cirrhosis is one in 20 pregnant women in the developing part of the world31 nevertheless, lack of reporting from these countries is one factor why true estimates of maternal mortality due to cirrhosis does not exist. Westbrook et al., also discussed a higher likelihood of detrimental pregnancy outcomes that may escalate with the severity of cirrhosis; such as preeclampsia and preterm birth.^32^ Therefore, medical experts are concerned over the conception in women suffering from cirrhosis. But with advancements in the healthcare and treatment of patients with liver diseases, the chances of fertility in women has also increased allowing an increased number of pregnancies in women with cirrhosis.^31^ Some recent studies on the relationship of pregnancy with hepatic diseases have reported appreciable outcomes of the mortality rates that are less than 2%.^32^ It is, therefore, less likely that the medical experts of the present time would discourage pregnancy.

### HCV Transmission from Mother to Child

Mother-to-Child Transmission (MTCT) of HCV is relatively uncommon, with a pooled infection rate ranging from 5.8 percent to 10.8 percent assessed in a data meta-analysis. It is one of the most serious outcomes of HCV infection in pregnancy, occurring in 5.8 percent (4.2 percent -7.8 percent) of mono-infection cases and 10.8 percent (7.6-15.2 percent) of maternal HIV-HCV co-infection cases.15 HCV MTCT, also known as vertical transmission of the virus, can occur throughout the intrauterine, intrapartum, and postnatal periods. Although the risk of vertical transmission of hepatitis-C in HCV negative RNA mothers was shown to be minimal in the research, intermittent viraemia in expectant women remained a significant risk for HCV vertical transmission. MTCT of hepatitis-C was twice as likely in women with positive HCV RNA and HIV co-infection.^15^

According to a study, from 2007 to 2012, in Pakistan 260 children were born to HCV-infected women contracted the infection vertically^33^ though the study highlighted over-estimation due to some limitations in accessing representative data from all regions of Pakistan. After vertically acquiring the HCV infection, it was observed that in children, the virus was spontaneously cleared in 25 to 40 percent within the first four years of their life which is a slightly higher rate of viral clearance than is reported for adults. The premeditated clearance rate of the MTCT-HCV infection may be attributed to several hosts and biological variables such as the IL28B gene, natural cell killers, cell cytolytic activity, and viral features such as HCV genotype are all involved.

There is limited evidence that the risk of perinatal transmission of HCV in children rises with greater maternal HCV viral load, longer labor length, use of amniocentesis or fetal scalp monitoring, and protracted membrane rupture.^13^ Chronic HCV infection in children is indolent, meaning that it develops slowly, most likely 10 years after infection beginning, with no acute problems compared to adults, and is frequently asymptomatic in early childhood. However, the illness is extensively documented in children who have had the infection for a longer period of time and have a longer follow-up history.^34^

### Postnatal Considerations and Fetal and Neonatal Impacts of HCV

The HCV has several detrimental impacts on the developing fetus in the HCV-infected intrauterine environment. Pregnancy complications caused by HCV, including as gestational hyperglycemia and hypertension, are substantially more prevalent in carrier mothers than uninfected ones. Moreover, the preterm birth rate (delivery before 37 weeks) is likely to increase to 13.1 percent compared to 8.8 percent in non-HCV infected mothers. Likewise, the prevalence of low birth weight (weight <2500g), small for gestational age (gestational weight less than 3 kg), and congenital anomalies escalates from 6.3 to 10.7 percent; 8.9 percent to 12.8 percent; and 3.4 to 4.6 percent in HCV-infected mothers in contrast to non-HCV infected mothers respectively.^30^

Additionally, another study has revealed that HCV transmission in the offspring of pregnant women with the virus may also be linked to an increased risk of gastrointestinal morbidities like gastroenteritis, colitis, and esophageal disease (adjusted HR = 2.26, 95% CI: 1.79-2.85; P < 0.001) as well as chronic respiratory diseases like asthma and obstructive sleep apnea (adjusted HR = 1.43, 95 percent CI 1.07-1.90, p = 0.015).17 Breast feeding can be continued in postpartum period and considered safe with negligible risk of HCV transmission.

### HCV Therapy during Pregnancy

Identification and medication of pregnant HCV-infected women are gaining attention globally in scientific and medical researchers as it cannot only cure the affected mother but has the potential to prevent the possibility of infection vertical transfer into offspring.^33^ Recently, the antiviral drug ribavirin that was initially used to treat Hepatitis-C is reported to cause detrimental effects in pregnant women. According to Sinclair Modin L et al., the medicine had severe teratogenic and embryotoxic effects in all animal species investigated in the study, and its usage in all types of HCV therapy, particularly for pregnant women, is not advised against.^35^

Furthermore, a novel technique to directly target viral suppression is now available; these are known as DAAs, direct-acting antivirals that are potentially way safer and do not include ribavirin. Direct acting antivirals (DAA) medications are oral, safe and effective treatment with short duration (12 weeks) causes the fast viral load decline with the onset of the treatment make the treatment more feasible and realistic but its use in pregnant patient’s is still not approved and various trials for its safety are ongoing. During the pregnancy period, it is easier to undergo testing and treatment of women in poor and middle-income nations who have HCV where usually irregular or incomplete postpartum follow-ups of mother and child are more common, thus this provides an opportunity for the proper healthcare management of both because during this period the pregnant women are more determined and motivated to ensure good health of themselves and the babies they are carrying.^36^

The American Association for the Study of Liver Disease (AASLD) and the Infectious Diseases Society of America (IDSA) proposed universal HCV screening in pregnant women in 2018. This recommendation was made a few years earlier by France and Pakistan, but it was not widely implemented by other organizations such as the Centers for Disease Control and Prevention (CDCP) and the Society for Maternal-Fetal Medicine (SMFM).^37^ If such screening procedures are applied during pregnancy, it is predicted it will discover roughly 300 infants with probable vertical HCV in the United States each year.^23^ Nonetheless, one restriction remains: pregnant women may face psychological stress as a result of the treatment’s inability to begin until the postpartum period. According to a recent poll, only 60% of women with HCV were willing to undertake HCV medication during pregnancy, despite being informed of all health and safety risks.^23^ Currently treatment in pregnancy is not recommended and screening and monitoring for complications remains the main stay.

### Impact of HCV on the Ability to Conceive

A significant relationship between hepatitis and women’s fertility was noted by several studies.38 These investigations have underlined the protective impact of fertile hormonal state on antiviral medication responsiveness and fibrosis development. However, near or immediately after menopause, an opposite result was observed, where rapid and unfavorable growth of the aforementioned conditions was observed. This observation, along with data collected from different countries in which profound consequences were observed on women’s reproductive function due to hepatitis, proved that a strong relationship between HCV infections can be established with the reproductive status of women. Moreover, in another study performed in Italy, the relationship of HCV with women’s fertility was established with Anti-Mullerian Hormone (AMH). AMH is generated by growing ovarian follicle cells and begins to diminish around one year before follicle-stimulating hormone levels rise. As a result, the AMH level may be a possible indicator of a woman’s reproductive capabilities. Its decreasing or lower level in women implies ovarian senescence.39 The study found that HCV+ women had considerably lower levels of AMH and a greater miscarriage rate than the uninfected control group. Furthermore, HCV+ women showed lower AMH levels than controls, indicating general ovarian physiology impairment.^39^

To date, there is no successful vaccine available to limit the spread of the Hepatitis-C virus and prevention is the key. Direct-acting antiviral agents can be a possible solution for preventing the MTCT in future. In some small-scale initial study, Sofosbuvir and Ledipasvir are the safest options to provide women during pregnancy. However, there is no large randomized control trials available that describes the safety of the new direct-acting antiviral agents in pregnancy for treating Hepatitis-C virus.26 Therefore, in order to prevent vertical transmission, it is necessary to treat the Hepatitis-C virus infection before the conception or planning a pregnancy.

## DISCUSSION

Chronic Hepatitis-C infection is bound to exacerbate transaminase levels in addition to causing substantial histopathological changes in the liver, while this degeneration and inflammation are noted to exacerbate during the postpartum period in women with advanced liver disease. Given the body of evidence highlighting these changes, it is suggested that pregnancy can worsen liver inflammation which may continue to exacerbate even during the postpartum period due to several immunological modulations and physiological changes in the body, which could also negatively influence the course of the disease.

It is recommended that modeling studies should be used to estimate the true prevalence of the HCV infection amongst women of reproductive age as the current estimates are incapable of measuring the actual burden of the disease, resulting in many women getting diagnosed with HCV virus during pregnancy due to antenatal screening in some geographic locations where HCV screening during pregnancy is mandatory. Moreover, in a setting where antenatal screening for HCV is not universal, preconception testing and treatment interventions to reduce the disease burden are imminent. Implementing these strategies will also reduce the MTCT of HCV in high-risk settings thereby reducing the cost of pediatric Hepatitis-C management. Such interventions, at the population level, may encompass addressing HCV transmission routes including screening of women of childbearing age, antenatal management of HCV, and linking it with postpartum care.

Lastly, in Pakistan, fulfilling the unmet need for family planning, specifically among women with HCV-positive antibodies, will be a potential intervention to limit the burden and consequences of pregnancy in HCV-positive women. Even while there isn’t an immediate medical emergency, the potential for a cure and the necessity for further steps to contain the HCV pandemic should serve as a call to action. Future research should confirm that DAAs may be used safely and successfully in expecting mothers and that healthcare professionals are able to intervene when necessary. Viral hepatitis is still a major pandemic that needs serious medical attention. Despite the restricted availability of medical resources, health officials should find a method to give viral hepatitis sufferers priority. Otherwise, we run the danger of swamping already overburdened healthcare facilities with complex liver cases.

## CONCLUSION

Globally, a large number of childbearing-age women become affected by HCV every year and vertical transmission of HCV is still a serious public health concern. The immunological changes during pregnancy strongly influence the progression of HCV infection especially during the third trimester and provide opportunistic conditions for the virus to disseminate. Lastly, the pregnancy outcomes of HCV infection are still ambiguous especially in Pakistan where the lack of data and effective reporting of such cases is still a limitation, therefore, we recommend that every pregnancy must be considered as high-risk until the mother screens negative for HCV antibodies. This approach will benefit both mother and the child by reducing the negative implications of an unattended high-risk pregnancy with an additional risk of gestational diabetes, preterm births, hypertension, and low gestational weight alongside viremia. With the change in HCV treatment and management modality from highly complicated interferon therapy’ to an efficient and more tolerable direct-acting antiviral therapy, it would be safer to manage and investigate pregnancy outcomes in HCV-positive women.
